# Theoretical Analysis and Design of Ultrathin Broadband Optically Transparent Microwave Metamaterial Absorbers

**DOI:** 10.3390/ma11010107

**Published:** 2018-01-11

**Authors:** Ruixiang Deng, Meiling Li, Badar Muneer, Qi Zhu, Zaiying Shi, Lixin Song, Tao Zhang

**Affiliations:** 1 Key Laboratory of Inorganic Coating Materials CAS, Shanghai Institute of Ceramics, Chinese Academy of Sciences (SICCAS), Shanghai 200050, China; dengrx@mail.ustc.edu.cn (R.D.); shizaiying@student.sic.ac.cn (Z.S.); lxsong@mail.sic.ac.cn (L.S.); 2 Department of Electronic Engineering and Information Science, University of Science and Technology of China (USTC), Hefei 230027, China; pheelin@ustc.edu.cn (M.L.); badar.muneer@faculty.muet.edu.pk (B.M.); 3 University of Chinese Academy of Sciences (UCAS), Beijing 100049, China; 4 Department of Telecommunication Engineering, Mehran University of Engineering & Technology, Jamshoro 72062, Pakistan

**Keywords:** metamaterial, microwave absorber, controllable design approach, optical transparency, absorption mechanism, broadband absorption

## Abstract

Optically Transparent Microwave Metamaterial Absorber (OTMMA) is of significant use in both civil and military field. In this paper, equivalent circuit model is adopted as springboard to navigate the design of OTMMA. The physical model and absorption mechanisms of ideal lightweight ultrathin OTMMA are comprehensively researched. Both the theoretical value of equivalent resistance and the quantitative relation between the equivalent inductance and equivalent capacitance are derived for design. Frequency-dependent characteristics of theoretical equivalent resistance are also investigated. Based on these theoretical works, an effective and controllable design approach is proposed. To validate the approach, a wideband OTMMA is designed, fabricated, analyzed and tested. The results reveal that high absorption more than 90% can be achieved in the whole 6~18 GHz band. The fabricated OTMMA also has an optical transparency up to 78% at 600 nm and is much thinner and lighter than its counterparts.

## 1. Introduction

Optically transparent microwave absorbers are a kind of absorbers that can not only absorb the microwave energy but also transmit the visible light. There are urgent needs to investigate the optically transparent microwave absorbers in both civil and military fields because of their unique properties and applications. Usually, it is not easy to realize optical transparency and microwave absorption simultaneously by coating materials as they are often contradictory [1,2]. Traditional optically transparent microwave absorbers reported in references are in the form of the Salisbury screen or Jaumann screen. However, they all have deficiencies such as narrow absorption band or thicker structures [3,4,5,6].

Metamaterials [7], consisting of artificial periodic subwavelength ‘atoms’ with the exotic electromagnetic properties that cannot be not found in nature materials [7,8,9], provide a brand new thought on designing microwave absorbers [7,8,9,10,11,12,13,14,15,16,17]. Microwave metamaterial absorbers (MMAs) possess a tremendous ability to achieve broadband absorption [10,11,13], ultrathin thickness [11], and light weight [13,14,15] at the same time. It is also of great potential to integrate many other functions [13,16], for instance, transparence to visible light. A great deal of research works have focused on MMAs and contributed a lot lately. Creative metasurfaces have been investigated to achieve novel MMAs. Features of complex plane geometries were studied to increase resonate frequencies [9,17] by numbers of researchers. Ding et al. [10] designed a three-dimensional quadrangular frustum pyramids array as MMA to achieve high absorption in an 8–14 GHz band. Inspired by “moth-eye” structures, Bychanok et al. [14,15] introduced 2D packed carbon sphere arrays to design bionic anti-reflective metasurfaces for MMA. However, the majority of the existing MMAs is monofunctional, opaque or cannot work in a broad band. Only a few efforts on optically transparent microwave metamaterial absorbers (OTMMAs) have been reported in recent years [18,19]. Nevertheless, they are not able to meet the needs of applications well. 

Furthermore, recent research works have reported many novel structure designs and analyzed the fantastic electromagnetic responses of their designs, which contributed valuable highlights to the development of metamaterial microwave absorbers. However, it is not easy for researchers to design another geometry of a metamaterial based on their conclusions. Practically viable design methods for MMAs are still unavailable, which principally increases challenges to the development of ultrathin broadband OTMMAs.

Therefore, absorption mechanisms of MMAs have been heatedly investigated. The equivalent medium model and the equivalent circuit model are two common methods used to analyze the working mechanisms of MMAs. The equivalent medium model [7,8,14,17,20,21] is based on electromagnetic theory. Even though this model can precisely predict the response process and mechanisms of MMAs, only few researchers opt for this design model due to complexities involved in solving Maxwell’s equations. The equivalent circuit model [12,13,22,23], based on the transmission line theory, is another widely accepted analysis tool. The equivalent circuit model is an abstract physical description of metamaterial absorber, showing the working mechanisms of metamaterial from a special view. The method is operational and can sententiously describe the working mechanisms of MMAs.

In this work, the equivalent circuit model of MMA is utilized as springboard to navigate the design of the geometric structure. Proceeding from the impedance matching principle, the equivalent circuit model of the MMAs is systematically analyzed. Through strict mathematical calculations, expressions of theoretical equivalent resistance as well as the quantitative relation between the equivalent capacitance and inductance at specific absorption frequency are obtained. In addition, the characteristics of equivalent resistance varied with the absorption frequency are explored. Based on these theoretical studies, we propose an effective approach to designing the OTMMAs with two controllable absorption peaks or a broad bandwidth for applications on stealth of the metal target. In order to validate the theoretical study, an ultrathin lightweight OTMMA operating in a 6–18 GHz band was designed, fabricated, tested and discussed.

## 2. Theoretical Analysis and Design

### 2.1. Mathematical Analysis Based on Transmission Line Theory

**Figure 1:**
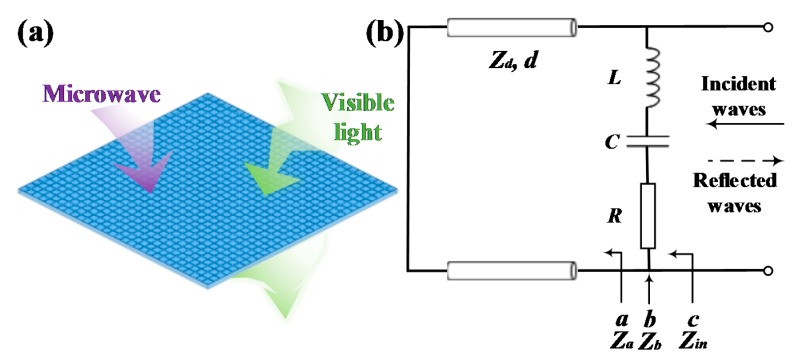
(**a**) The concept of OTMMA; (**b**) the equivalent circuit model of metamaterial absorbers.

The metamaterial absorber is comprised of a dielectric substrate with a metasurface on the top. The surface of metal target can be regarded as groundplane. As simplification, it is assumed that the dielectric substrate is lossless and nonmagnetic and the absorber is irradiated by normally incident plane waves. The lossy metasurface is responsible for energy consumption. Conforming to the response of MMA in an external electromagnetic field, a general equivalent circuit of MMA is established in Figure 1b. The dielectric substrate is deemed as a transmission line with characteristic impedance *Z_d_*. The metasurface is modeled as cascaded resistance *R*, inductance *L* and capacitance *C*.

When an absorber is exposed to external normally incident microwaves, no transmission takes place due to the metal target. The microwave is either reflected or absorbed. Here, the absorption ratio can be used to evaluate the performance of the absorber, namely,

(1) $$Absorption=1-Reflection=1-{\left|\frac{Z_{in}-Z_{0}}{Z_{in}+Z_{0}}\right|}^{2},$$

where *Z*_0_ = 377 Ω is the characteristic impedance of free space and *Z_in_* is the input impedance of the absorber.

Looking from port ‘*a*’ towards the metal target, the input impedance *Z_a_* is [24]

(2) $$Z_{a}=j\frac{Z_{0}}{\sqrt{\varepsilon_{r}}}\tan\frac{2\pi f\sqrt{\varepsilon_{r}}d}{c},$$

where *j* is the imaginary unit, *ε_r_*, *d* are the relative permittivity and thickness of the dielectric substrate, respectively, *f* is the frequency of the incident electromagnetic waves and *c* is the velocity of light. Meanwhile, the impedance of the metasurface *Z_b_* can also be written as

(3) $$Z_{b}=R+j\left(2\pi fL-\frac{1}{2\pi fC}\right).$$

Hence, the overall input impedance *Z_in_* is given by 

(4) $$Z_{in}=\frac{Z_{a}Z_{b}}{Z_{a}+Z_{b}}.$$

In order to match with the free space, the total input impedance Zin must be equal to Z0. Combining Equations (1)–(4), the constraints can be expressed as follows:

(5) $$\{\begin{array}{c} \frac{R}{R^{2}+{\left(2\pi fL-\frac{1}{2\pi fC}\right)}^{2}}=\frac{1}{Z_{0}}\\ \frac{\left(2\pi fL-\frac{1}{2\pi fC}\right)}{R^{2}+{\left(2\pi fL-\frac{1}{2\pi fC}\right)}^{2}}=\;\frac{\sqrt{\varepsilon_{r}}}{Z_{0}}\cot\left(\frac{2\pi f\sqrt{\varepsilon_{r}}d}{c}\right). \end{array}$$

From Equation (5), two important relationships can be obtained:

(6) $$R=\frac{Z_{0}\tan^{2}\left(\frac{2\pi f\sqrt{\varepsilon_{r}}d}{c}\right)}{\varepsilon_{r}+\tan^{2}\left(\frac{2\pi f\sqrt{\varepsilon_{r}}d}{c}\right)},$$

(7) $$\left(2\pi fL-\frac{1}{2\pi fC}\right)=-\frac{\sqrt{\varepsilon_{r}}Z_{0}\tan\left(\frac{2\pi f\sqrt{\varepsilon_{r}}d}{c}\right)}{\varepsilon_{r}+\tan^{2}\left(\frac{2\pi f\sqrt{\varepsilon_{r}}d}{c}\right)}.$$

**Figure 2:**
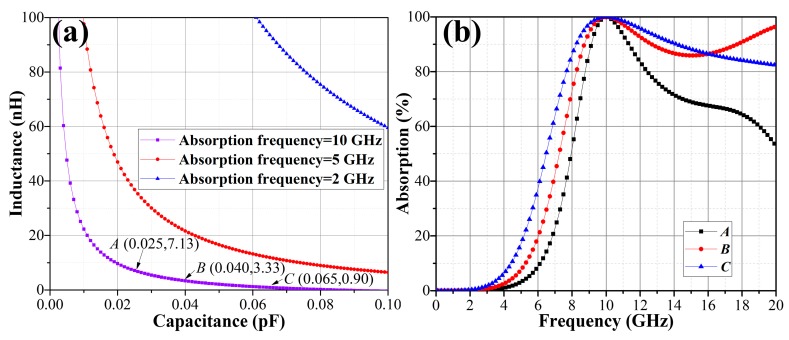
(**a**) the *L*–*C* relationship curves at 2 GHz, 5 GHz and 10 GHz: dielectric thickness *d* = 3 mm and relative permittivity *ε_r_* = 2.5; (**b**) the absorption curves of three design examples with the design parameters presented in Table 1.

Equation (6) provides the theoretical value of equivalent resistance needed at specific absorption frequency. The theoretical equivalent resistance remains constant for given dielectric parameters and absorption frequency. Equation (7) suggests the quantitative relation between the equivalent inductance *L* and equivalent capacitance *C*. Moreover, the absorption frequency is dependent on *L* and *C*. For given *d*, *ε_r_* and absorption frequency, the *L*–*C* relation is also definite. The *L*–*C* relation curves under different absorption frequencies with *ε_r_* = 2.5 and *d* = 3 mm are shown in Figure 2a. There are countless (*L*, *C*) solutions available at a single absorption frequency. Three points *A*, *B* and *C* on the locus of 10 GHz curve are chosen for the validation. The design parameters and absorption performances are displayed in Table 1. The results in Figure 2b reveal that the design of OTMMAs’ equivalent circuit at any single absorption frequency can be achieved based on Equations (6) and (7).

### 2.2. Frequency-Dependent Characteristics of Equivalent Resistance

For a given dielectric substrate, the theoretical equivalent resistance is a function of the absorption frequency. In order to find out the accurate frequency-dependent characteristics of the theoretical equivalent resistance, the first derivative of Equation (6) with respect to frequency *f* is taken:

(8) $$\frac{dR}{df}=\frac{2Z_{0}\varepsilon_{r}\tan\left(\frac{2\pi f\sqrt{\varepsilon_{r}}d}{c}\right)\sec^{2}\left(\frac{2\pi f\sqrt{\varepsilon_{r}}d}{c}\right)}{{\left[\varepsilon_{r}+\tan^{2}\left(\frac{2\pi f\sqrt{\varepsilon_{r}}d}{c}\right)\right]}^{2}}.$$

The frequencies at which the theoretical equivalent resistance reach the extreme points can be obtained when Equation (8) equals zero:

(9a) $$f_{min}=\frac{\left(N-1\right)c}{2\sqrt{\varepsilon_{r}}d},$$

(9b) $$f_{max}=\frac{c}{4\sqrt{\varepsilon_{r}}d}+\frac{\left(N-1\right)c}{2\sqrt{\varepsilon_{r}}d},$$

where *N* is equal to 1, 2, 3 ….

The theoretical equivalent resistance reaches the minimum *R* = 0 Ω when the absorption frequency is equal to *f_min_*, whereas there is a maximum *R* = 377 Ω as the absorption frequency reaches *f_max_*. Equations (10) and (11) can be validated through the mathematical proof:

(10) $$R\left(f+\frac{c}{2\sqrt{\varepsilon_{r}}d}\right)=R\left(f\right),$$

(11) $$R\left(2f_{max}-f\right)=R\left(f\right).$$

The first one interprets that the theoretical equivalent resistance value changes periodically with the absorption frequency and the period *T* is *c*/(2$\sqrt{\varepsilon_{r}}$*d*). For ease of the following discussion, the distance between two adjacent minimum frequency points is taken as one time period *N*. In addition, Equation (11) states that the theoretical equivalent resistances are symmetrically distributed about the maximum frequencies *f_max_* in every period.

**Figure 3:**
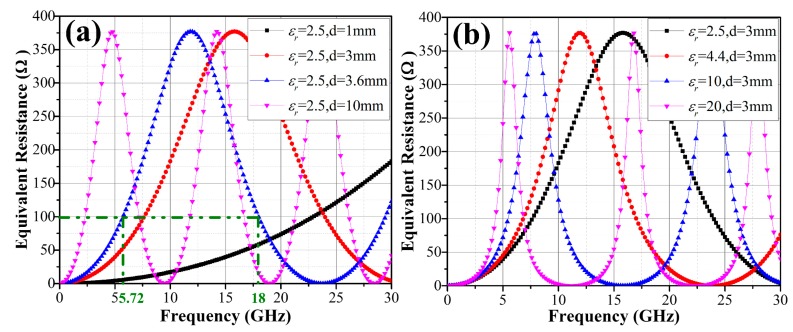
(**a**) The *R*-*f* curves with fixed relative permittivity *ε_r_* = 2.5 but different thickness *d*; (**b**) the *R*-*f* curves with fixed thickness *d* = 3 mm but different relative permittivity *ε_r_*.

Periodicity and axial symmetry are two distinguishing frequency-dependent characteristics of the theoretical equivalent resistance and they are correlated with the parameters of the dielectric substrate. Figure 3a,b shows the frequency-dependent characteristics curves of equivalent resistance with different thickness *d* and different relative permittivity *ε_r_*, respectively. It is worth noting that the value varies from 0 to 377 Ω periodically regardless of the values of thickness and relative permittivity. The period *T* decreases with the increasing of thickness *d* and relative permittivity *ε_r_*. According to Equations (9)–(11), the symmetric equivalent resistance curve can be centered at any desired frequency and the period *T* can also be adjusted by selecting proper *d* and *ε_r_*. In this manner, frequency-dependent characteristics of equivalent resistance can be applied to effectively achieve a precise design of OTMMAs with two absorption peaks or a broad absorption band.

### 2.3. Tunable Design of Two-Peak OTMMAs

The impedance matching to free-space impedance is achievable at more than one frequency point from the frequency distribution in Figure 3, owing to the periodic and symmetric distribution of absorption frequencies about *f_min_* or *f_max_* for a fixed theoretical equivalent resistance when the dielectric substrate is given.

Before the equivalent circuit for OTMMA is designed, it is necessary to select a collection of alternate optically transparent dielectric materials with various permittivity. To design an absorber working at two frequencies *f*_1_ and *f*_2_, with maximum thickness *d*, the theoretical equivalent resistance must be the same at both of the frequencies. This can be achieved by adjusting the frequency point *f_max_* to be at the center of the two absorption frequencies *f*_1_ and *f*_2_. It is not advisable to symmetrize the two frequencies about *f_min_* because there may be no solution for (*L*, *C*) in most cases.

**Figure 4:**
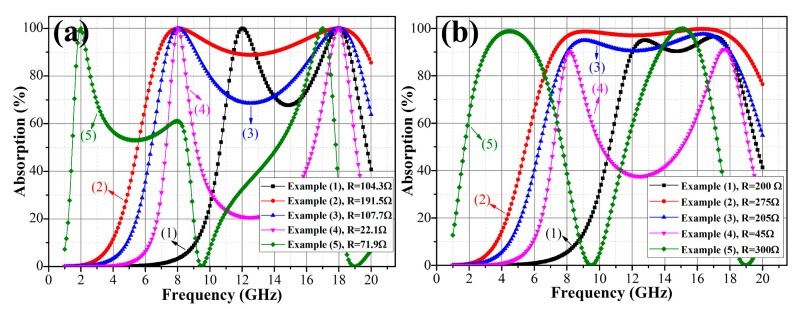
The absorption performances of example absorbers with (**a**) *R* calculated using Equation (6) and (**b**) optimized *R*.

Using Equation (9b), the minimum value of relative permittivity *ε_r_min_* corresponding to the required thickness can be calculated. The suitable dielectric material can then be selected according to the calculated *ε_r_min_* and the actual thickness of the absorber can also be determined. Once the two absorption frequencies and parameters of dielectric substrate are determined, two *L*-*C* relation equations are obtained to find a unique solution for *L* and *C*. In Figure 4a, several examples of absorbers having two adjustable absorption frequencies *f*_1_ and *f*_2_ or different thickness *d* are designed. The parameters of the each absorber are provided in Table 2. 

### 2.4. Tunable Design of Broadband OTMMAs

The design of a broadband OTMMA begins with designing a two-peak OTMMA according to Section 2.3 with the *f*_1_ and *f*_2_ designed to be approximately equal to the lower and upper cut-off frequencies of the broadband absorber, respectively. It has been discussed in Section 2.1 that equivalent inductance *L* and equivalent capacitance *C*, which mainly account for impedance matching, determine the absorption frequencies *f*_1_ and *f*_2_, while equivalent resistance *R* is responsible for energy dissipation and is influential in the impedance match. Thus, a broadband behavior of absorbers can be realized by optimizing the equivalent resistance *R* while keeping *L* and *C* unchanged. Figure 4b shows the design examples of absorbers using the same dielectric substrate, inductance *L* and capacitance *C*, but a larger value of equivalent resistance *R*. Compared with the results in Figure 4a, the broadband behavior of absorbers having cut-off frequencies close to *f*_1_ and *f*_2_ can be observed. 

The reason can be explained by the lower quality factor *Q* resulted from the increasing value of equivalent resistance *R*. The *Q* factor of an absorber is defined as

(12) $$Q=2\pi f\frac{P_{s}}{P_{l}}=\frac{f}{\Delta f},$$

where *f* is absorbing frequency, *P_s_* and *P_l_* are stored energy and consumed energy, respectively, and Δ*f* denotes the bandwidth of the absorber. It is obvious that the bandwidth will be broadened with the appropriate increasing of equivalent resistance *R*, which leads to more energy dissipation. However, it not advisable to overly raise the value of equivalent resistance *R* since impedance mismatch will occur in this circumstance.

It is worth mentioning that the two frequencies *f*_1_ and *f*_2_ must lie within the same period *N* so as to achieve a broadband absorption. If there exists a frequency (*f_min_*) where the theoretical equivalent resistance becomes zero (*R* = 0) between the frequencies *f*_1_ and *f*_2_, the total mismatch will occur, making it impossible to design a wideband absorber. For example, on curve-(5) in Figure 4, total reflections occur at 9.5 GHz and 19 GHz, where *R* becomes zero (see the pink curve in Figure 3a). To avoid a large value of relative permittivity *ε_r_* and thicknesses *d* and small value of theoretical resistance *R*, it is advisable to place the two frequencies in the first period *N* = 1.

**Figure 5:**
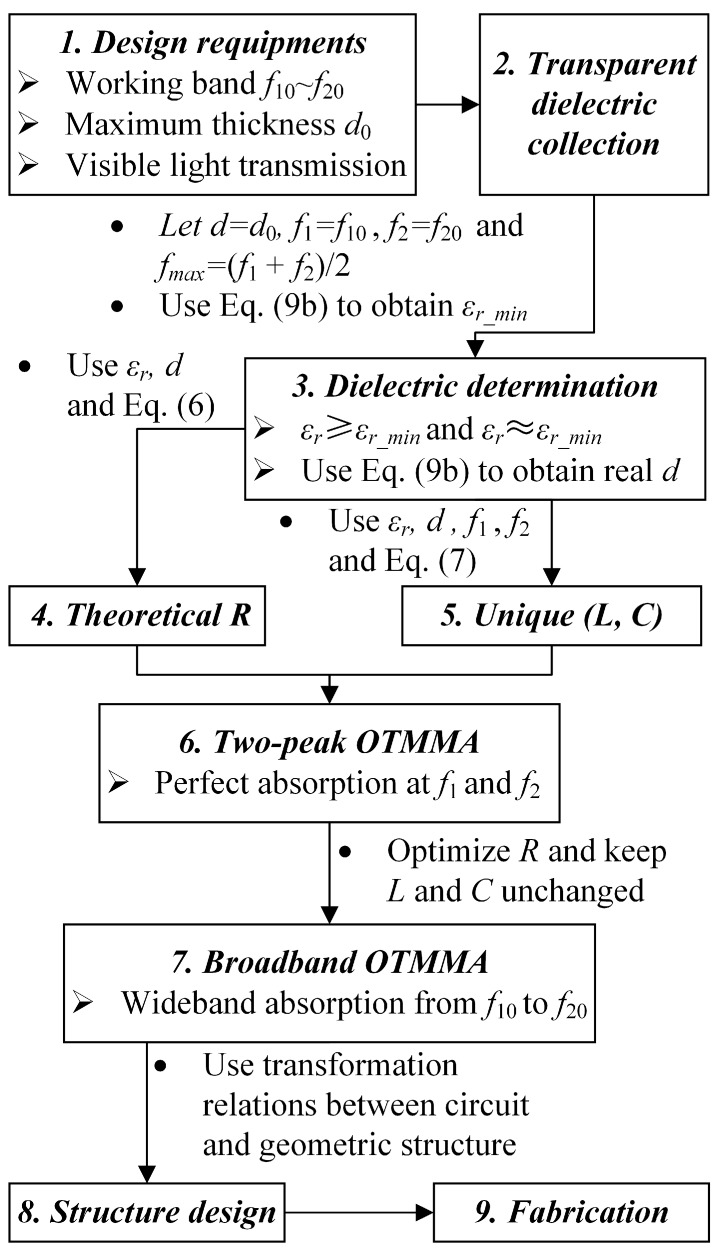
The proposed controllable design approach to OTMMAs.

Theoretically, the larger the relative permittivity, the smaller the thickness, according to Equation (9). As seen in curve-(2), (3) and (4) in Figure 4, large relative permittivity can make thickness smaller. However, unduly large value of relative permittivity may not be favorable to design a wideband absorber, since a large difference between *ε_r_* and *ε*_0_ (free-space permittivity) results in a huge mismatch [25]. On the basis of the discussions above, an effective design approach to OTMMAs is proposed in Figure 5. The design of single-layer OTMMAs with a tunable single narrow band, dual narrow bands or broadband absorption can be realized through the approach.

## 3. Verification and Experiment

**Figure 6:**
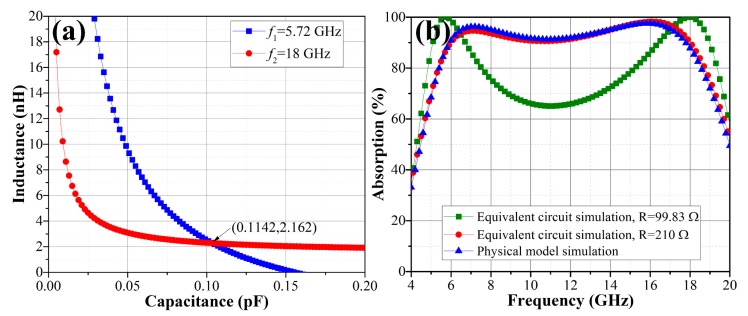
(**a**) The *L*-*C* relationship curves at 5.72 GHz and 18 GHz when *d* = 3.6 mm and *ε_r_* = 2.5; (**b**) the simulated absorption performances using ADS and HFSS under normally incident plane waves.

In order to validate the proposed design approach, an OTMMA operating in a 6–18 GHz band is developed. Polymethyl methacrylate (PMMA, *ε_r_* = 2.5) possesses advantages of high specific strength, high optical transparency as well as excellent chemical stability. Grounded PMMA with thickness *d* = 3.6 mm is adopted as the dielectric substrate. Thus, the *R*-*f* curve of the present substrate is given in Figure 3a with *ε_r_* = 2.5 and *d* = 3.6 mm. According to Figure 5, to design a wideband absorber operating in a 6–18 GHz band, first, a two-peak OTMMA exactly matched with the free-space at *f*_1_ = 5.72 GHz and *f*_2_ = 18 GHz is designed. Corresponding theoretical equivalent resistance for these two frequencies is 99.83 Ω, as marked in Figure 3a. Substituting the parameters in Equation (7), unique solution for *L* and *C* gives *L* = 2.162 nH and *C* = 0.1142 pF, as shown in Figure 6a.

Simulation of the equivalent circuit was carried out using Advanced Design System (ADS) with the above parameters and the absorption curve is displayed in Figure 6b, which is certainly consistent with the desired purpose. However, the absorption in 7–16.5 GHz band is still insufficient, which is not desirable for wideband requirement. To achieve the desired performance of a wideband absorber, equivalent resistance obtained for two-peak OTMMA (99.83 Ω) is adjusted to 210 Ω. The result in Figure 6b reveals that the absorber operates with absorption performance better than 90% from 6 GHz to 18 GHz.

**Figure 7:**
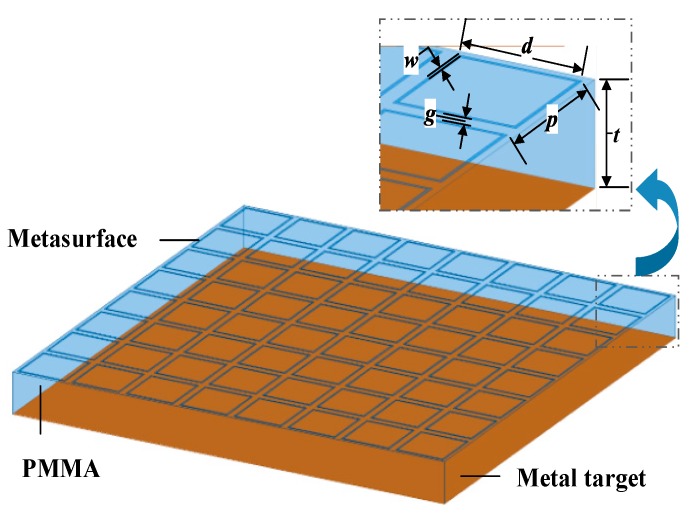
The structure of the designed OTMMA.

The physical model simulations under normally incident plane waves were then implemented in a High Frequency Structure Simulator (HFSS). Figure 7 describes the physical model of the designed OTMMA. In the physical model, the periodic centrosymmetric lossy square loop array is used as the metasurface. Four factors are taken into consideration. First, the OTMMA is insensitive to different polarized incident plane waves due to its centrosymmetric feature. Then, it is more simple and convenient to convert the equivalent circuit model into such a kind of metasurface. In addition, the square loop pattern can increase the optical transparency of OTMMA because of less area covered by conductive lossy films. Convenient fabrication procedure is another consideration. 

**Figure 8:**
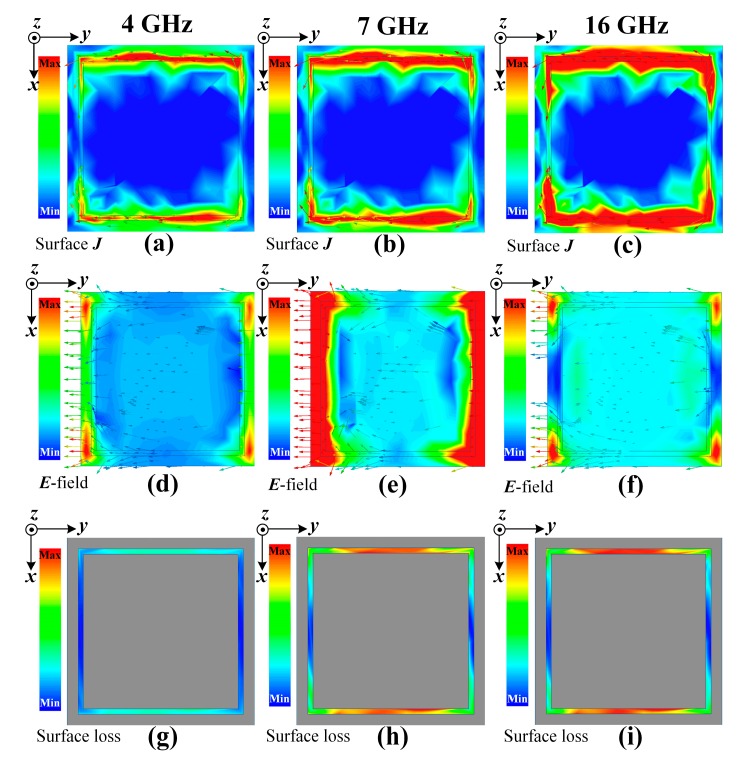
The (**a**–**c**) surface current density ***J*** distributions; (**d**–**f**) ***E***-field distributions and (**g**–**i**) surface loss density distributions of the designed OTMMA’s unit cell at 4, 7 and 16 GHz, respectively, at normal incident plane waves.

Transformation from an equivalent circuit model to the physical structure can be partially accomplished based on the method in [12,13,26,27], which illustrates the quantitive relationships between the equivalent lumped reactance and the periodic centrosymmetric lossy square loop array. Initial values of the dimensions can be calculated. Taking advantage of the designed structure’s electromagnetic responses in Figure 8, the dimensions of the initial structure can be further modified.

When the OTMMA is exposed to external normal incident plane waves, the surface current in the conductive metasurface will be induced due to the directional movement of the carriers in the action of the external electric field (***E***-field), as shown in Figure 8a–c. The induced surface current in the square loop can trigger the emergence of an equivalent inductance. Generally, the value of the inductance can be modified by adjusting the width *w* of the loop in this pattern. In addition, the current is discontinuous because of the existence of the gap in the direction parallel to the ***E***-field in each unit. The gap will give rise to the accumulation of charges in the arms perpendicular to the external ***E***-field, especially near the four corners, so that there occurs the maximum strength of an induced ***E***-field on the metasurface, as shown in Figure 8d–f. In this manner, the gap can be regarded as an equivalent capacitance, whose value is inversely proportional to the size of the gap. The final geometric values of the OTMMA are given in Table 3.

The ohmic loss caused by the metasurface can be deemed as an equivalent resistance. Comparing the ***E***-field distribution with the surface current density *J* distribution at the same frequency, it is found that most of the surface current is located in the position of the arms parallel to the ***E***-field. It means that these arms primarily contribute to electromagnetic energy absorption according to Joule’s law, *P_l_* = *I*^2^*R*_s_, where *I* is the current intensity and *R_s_* is the surface resistance of the metasurface. Hence, the effective area *A*_eff_ of two arms parallel to ***E***-field can thus be estimated as 1.64 mm^2^. The lumped resistance can be transformed to the surface square resistance using equation in [23], that is,

(13) $$R=R_{s}\frac{A}{A_{\mathrm{eff}}},$$

where *R* is the lumped resistance in the equivalent circuit and *A* is the area of the unit cell. The calculated surface resistance is 9.57 Ω/sq and the optimized value is 9 Ω/sq.

Absorption performance of modelled OTMMA in full wave simulation is presented in Figure 6b. It is seen that the proposed OTMMA has perfect absorption performance better than 90% in the whole range of 6–18 GHz. The highest absorption peaks occur at 7 and 16 GHz and the maximum absorption can reach up to as high as about 99.37%. The electromagnetic responses is researched in Figure 8 so as to have insight into the working mechanism of the OTMMA. Observed from Figure 8b,c, it can be deduced that the strong absorption at 7 and 16 GHz springs from the electric resonances since the current concentrated in the two parallel arms is always in the same direction, making it impossible to induce magnetic resonances. Comparing the surface current distributions at 4, 7 and 16 GHz in Figure 8a–c, the surface current density is much more intensive in the absorption band than that in the reflection band, which can explain how strong absorption happens. The evidence that more electromagnetic energy is dramatically consumed in the two arms parallel to ***E***-field at 7 and 16 GHz than that at 4 GHz in Figure 8g–i can also illustrate.

**Figure 9:**
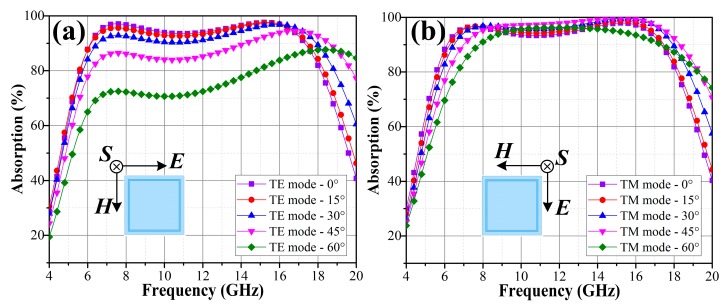
Simulated angular dependence of absorption performances of the OTMMA under different polarized incident waves: (**a**) TE and (**b**) TM waves.

Figure 9 compares the absorption performances of the OTMMA under obliquely incident TE waves and TM waves. 

No difference is observed between the normally incident TE and TM waves, which suggests the polarization insensitive feature of the OTMMA. Little deterioration in absorption performance is observed in both TE and TM waves when incident angle is blow 30°. However, as the incident angle of oblique waves increases from 30° to 60°, there is an obvious tendency of decreasing in absorption rate and shrinkage in bandwidth, while the absorption band also shifts to a higher frequency band. Considering oblique incidence, the input impedance $Z_{a}$ is [25]

(14) $$Z_{a}=j\frac{Z_{0}}{\sqrt{\varepsilon_{r}}}\tan\left(\frac{2\pi f\sqrt{\varepsilon_{r}}d}{c}\cos\theta \right),$$

where *θ* denotes the incident angle. It can be learnt that input impedance $Z_{a}$ decreases with the increasing of incident angle. Meanwhile, the projecting dimensions of the metasurface on the direction of the external ***E***-field decrease as well. The two factors lead to a gradual impedance mismatch between the absorber and the free space as incident angle increases, which accounts for degradation of absorption performance in the target band and shift behavior to a higher frequency band.

**Figure 10:**
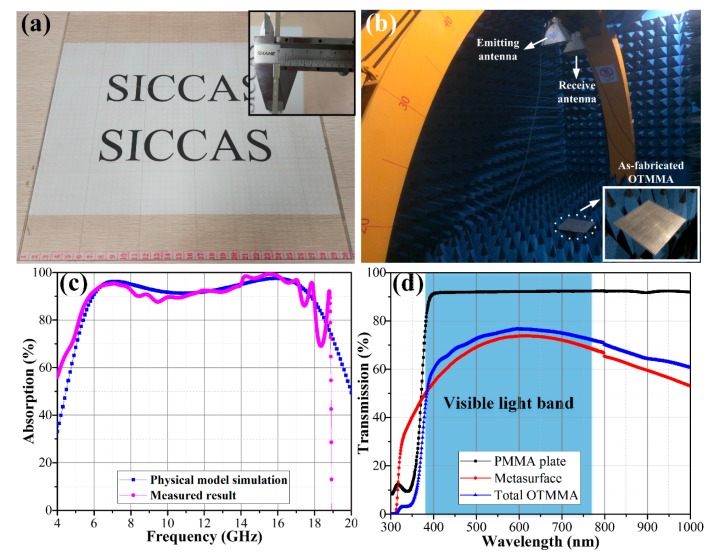
(**a**) front view and cross-section view (interpolation) of the fabricated large-scale OTMMA with 50 × 50 elements; (**b**) the RCS measurement system of OTMMA by arch test system; (**c**) the simulated and measured absorption performances of the OTMMA under normally incident plane wave; and (**d**) the tested transparency results of the OTMMA.

For further validation, a large-scale optically transparent absorber with 50 × 50 elements was fabricated, as shown in Figure 10a.

Radar cross section (RCS) reduction measurement was carried out by an arch test system as shown in Figure 10b. As the test results in Figure 10c point out, the absorption performance of the as-fabricated OTMMA is found to be dramatically better than 90% in the whole band of 6–18 GHz. The experimental result is in great agreement with the simulation results. 

In addition, an optical transparency test from the 300 to 1000 nm wavelength range was characterized. The result in Figure 10d demonstrates that the light transmission of the absorber in the whole visible light range is above 60% and the maximum transmission of 78% is obtained at 600 nm. It can be observed that light transmittance of the OTMMA (ITO-PET-PMMA) is higher than that of single metasurface (ITO-PET). The reason is attributed to the difference in the refractive index of the PMMA substrate and PET film. For incident light whose wavelength is *λ* = 600 nm, the corresponding refractive indexes of ITO, PET and PMMA are *n*_1_ = 1.49 [28], *n*_2_ = 1.66 [28] and *n*_3_ = 1.64 [29], respectively, while the refractive index of air is approximately equal to the vacuum space, *n*_0_ = 1. The energy attenuation coefficient in the PMMA is negligibly small. Thus, besides the part absorbed by ITO and PET, the light energy is either reflected or transmitted. The reflection coefficient of light at a boundary between two materials is [30]

(15) $$r=\frac{n_{i}-n_{m}}{n_{i}+n_{m}},$$

where *n_i_* and *n_m_* are the refractive indexes of material-*i* and material-*m*, respectively. It can be seen from Equation (15) that light energy is less transmitted and more reflected at the PET/air boundary than that at the PMMA/air and PMMA/PET boundaries because a larger gap in the refractive index exists between PET and air. Hence, there is a higher light transmittance of total OTMMA than that of the sole metasurface. In addition, the visible light transmission can be further improved by optimizing the fabrication procedure of the metasurface. A comparison of designed OTMMA with its counterparts is outlined in Table 4.

## 4. Materials and Methods

### 4.1. Simulation

Simulations of equivalent circuit were carried out using an Advanced Design System (ADS 2008, Agilent Technologies, Santa Clara, CA, USA). Full wave simulations of physical models were implemented in a High Frequency Structure Simulator (HFSS 2015, ANSYS, Inc., Canonsburg, PA, USA). In the full wave simulation, master and slave boundaries were used. A Floquet Port was selected as excitation.

### 4.2. Preparation

Highly transparent indium-tin-oxide (ITO) film with surface resistance of *R*_s_ = 9 Ω/sq was prepared on the polyethylene terephthalate (PET) substrate using a direct current (DC) magnetron sputtering method. The target material was composed of 10 wt % SnO_2_ and 90 wt % In_2_O_3_. Before deposition, the PET film was ultrasonically cleaned in alcohol and deionized water for 15 min, respectively. After that, PET film was dried by high purity nitrogen (purity 99.99%). The base pressure of the system was 8.0 × 10^−5^ Pa. Ar gas (purity 99.99%) then flowed into the chamber with a fixed flux of 100 sccm as glow discharge gas. The ceramic target was sputtered for 10 min to remove impurities before preparing. The ITO film was then deposited on PET film at a sputtering power of 120 W for 10 min with an oxygen flux of 2.5 sccm. The thickness of prepared ITO film and PET are 85 nm and 50 μm, respectively. Subwavelength patterns of the metasurface were etched through a laser lithography machine (SC-K600, Strong Laser Equipment Co. Ltd., Dongguan, China). The metasurface was manually stuck to a transparent PMMA plate by means of optically clear adhesive (OCA, 250U, Mitsubishi Chemical Corporation, Tokyo, Japan). The chemical composition of OCA is gummy PMMA.

### 4.3. Measurement

The RCS reduction measurement was conducted by an arch test method, one of the most efficient and widely accepted methods to assess the performance of microwave absorbers. The measurement in our manuscript was carried out based on Chinese test standard GJB2038A-2011 in a standardized microwave anechoic chamber. Double-ridged broadband horn antennas (BBHA 9120 D, Schwarzbeck Mess-Elektronik OHG, Schönau, Germany) and Vector Network Analyzer (ZVB 20, Rohde & Schwarz, Munich, Germany) were used. The commercial analysis software (version, WeiJue Electronic Science & Technology Co. Ltd., Shanghai, China) was adopted to obtain the reflection coefficient from RCS data. Then, the absorption coefficient was finally calculated according to Equation (1). In the test, a 300 × 300 × 2 mm^3^ Aluminum plate was used as a stealth object. Both the RCS of sole object and object covered by the OTMMA were tested under normally incident, horizontally polarized waves in a frequency range of 2–18 GHz.

Optical transparency of the fabricated OTMMA was characterized by an ultraviolet-visible-near infrared (UV-Vis-NIR) spectrophotometer (Agilent Cary 5000, Agilent Technologies, Santa Clara, CA, USA) from 300 to 1000 nm wavelength range.

## 5. Conclusions

In this paper, an equivalent circuit model is adopted as springboard to navigate the design of OTMMA. The quantitative relationship between equivalent inductance and capacitance as well as the mathematical expression of theoretical equivalent resistance at a specific absorption frequency are derived. It has been demonstrated that the periodic and symmetric distribution of theoretical equivalent resistance over absorption frequency can be controlled or adjusted by selecting a proper dielectric substrate. Based on the theoretical studies, an effective approach to design single peak, two peak and broadband OTMMAs is proposed. The effects of dielectric permittivity on the thickness and performance of OTMMA are studied in detail. It is concluded that higher dielectric permittivity can help to reduce the thickness of the absorber. However, unduly large permittivity can also result in impedance mismatch and realization difficulties. The proposed approach is validated experimentally by fabricating a wideband OTMMA. The results indicate that the absorber can work steadily in a 6–18 GHz band with high optical transparency of 78% at 600 nm. The designed OTMMA has the properties of compact ultra-thin structure, optical transparency, wide bandwidth and design flexibility.

## Figures and Tables

**Table 1:** The design parameters of three examples in Figure 2b and their absorption performances. The *R* is calculated from Equation (6) by replacing *ε_r_*, *d* and *f* with 2.5, 3 mm and 10 GHz, respectively. The *L* and *C* are extracted from the purple curve in Figure 2a.

No.	*R* (Ω)	*C* (pF)	*L* (nH)	Bandwidth of Absorption Better Than 90% (GHz)
*A*	182.95	0.025	7.13	2.2
*B*	182.95	0.040	3.33	4.1
*C*	182.95	0.065	0.90	5.9

**Table 2:** The parameter details of design examples in Figure 4.

No.	*ε_r_*	*d* (mm)	*f*_1_ (GHz)	*f*_2_ (GHz)	*R* in Figure 4a (Ω)	*R* in Figure 4b (Ω)
(1)	25.0	1	12	18	104.3	200
(2)	2.08	4	8	18	191.5	275
(3)	5.32	2.5	8	18	107.7	205
(4)	33.28	1	8	18	22.08	45
(5)	2.5	10	2	17	71.88	300

**Table 3:** Geometric parameters of each unit cell in the designed OTMMA in Figure 7.

*d* (mm)	*p* (mm)	*t* (mm)	*w* (mm)	*g* (mm)
5.3	6	3.6	0.17	0.7

**Table 4:** Comparison on absorption performance between OTMMA in this work and previous works.

Absorber	Absorption Band above 90% (GHz)	Relative Bandwidth ^1^	Thickness (mm)	Relative Thickness ^2^	Optical Transparency
Ref. [4]	8.3~11.6	1.40	24.56	0.679	Not given
Ref. [5]	125~165	1.32	1.3	0.542	80~85%
Ref. [6]	About 8.7~11.2	1.28	3.6	0.104	80~85%
Ref. [18]	0.915~0.928	1.01	5.1	0.015	About 75%
Ref. [19]	9.6~13.9	1.45	2.1	0.068	Metal mesh-96.3% ITO sheet-90% Total-not given
This work	6~18	3	4.0	0.080	78% at 600 nm Total-above 60%

^1^ Relative bandwidth is defined as the ratio of the highest frequency and the lowest frequency in the band when absorption is better than 90%; ^2^ Relative thickness is defined as the ratio of the thickness and wavelength of the lowest absorption frequency when absorption is better than 90%.

## References

[1] Pullar R.C. (2012). Hexagonal ferrites: A review of the synthesis, properties and applications of hexaferrite ceramics. Prog. Mater. Sci..

[2] Liu P.J., Yao Z.J., Zhou J.T., Yang Z.H., Kong L.B. (2016). Small magnetic Co-doped NiZn ferrite/graphene nanocomposites and their dual-region microwave absorption performance. J. Mater. Chem. C.

[3] Takizawa K., Hashimoto O. (1999). Transparent wave absorber using resistive thin film at V-band frequency. IEEE Trans. Microw. Theory Tech..

[4] Haruta M., Wada K., Hashimoto O. (2000). Wideband wave absorber at X frequency band using transparent resistive film. Microw. Opt. Technol. Let..

[5] Wu B.A., Tuncer H.M., Naeem M., Yang B., Cole M.T., Milne W.I., Hao Y. (2014). Experimental demonstration of a transparent graphene millimetre wave absorber with 28% fractional bandwidth at 140 GHz. Sci. Rep..

[6] Grande M., Bianco G.V., Vincenti M.A., de Ceglia D., Capezzuto P., Petruzzelli V., Scalora M., Bruno G., D’Orazio A. (2016). Optically transparent microwave screens based on engineered graphene layers. Opt. Express.

[7] Schurig D., Mock J.J., Justice B.J., Cummer S.A., Pendry J.B., Starr A.F., Smith D.R. (2006). Metamaterial electromagnetic cloak at microwave frequencies. Science.

[8] Landy N.I., Sajuyigbe S., Mock J.J., Smith D.R., Padilla W.J. (2008). Perfect metamaterial absorber. Phys. Rev. Lett..

[9] Watts C.M., Liu X.L., Padilla W.J. (2012). Metamaterial Electromagnetic Wave Absorbers. Adv. Mater..

[10] Ding F., Cui Y.X., Ge X.C., Jin Y., He S.L. (2012). Ultra-broadband microwave metamaterial absorber. Appl. Phys. Lett..

[11] Liu Y.H., Gu S., Luo C.R., Zhao X.P. (2012). Ultra-thin broadband metamaterial absorber. Appl. Phys. A-Mater..

[12] Liu T., Kim S. S. (2016). Design of wide-bandwidth electromagnetic wave absorbers using the inductance and capacitance of a square loop-frequency selective surface calculated from an equivalent circuit model. Opt. Commun..

[13] Li M.L., Yi Z.X., Luo Y.H., Muneer B., Zhu Q. (2016). A novel integrated switchable absorber and radiator. IEEE Trans. Antennas Propag..

[14] Bychanok D., Li S., Sanchez-Sanchez A., Gorokhov G., Kuzhir P., Ogrin F.Y., Pasc A., Ballweg T., Mandel K., Szczurek A. (2016). Hollow carbon spheres in microwaves: Bio inspired absorbing coating. Appl. Phys. Lett..

[15] Bychanok D., Li S., Gorokhov G., Piasotski K., Meisak D., Kuzhir P., Burgess E.A., Gallagher C.P., Ogrin F.Y., Hibbins A.P. (2017). Fully carbon metasurface: Absorbing coating in microwaves. J. Appl. Phys..

[16] Alvarez H.F., Gomez M.E.D., Las-Heras F. (2015). A thin c-band polarization and incidence angle-insensitive metamaterial perfect absorber. Materials.

[17] Cheng Y.Z., Cheng Z.Z., Mao X.S., Gong R.Z. (2017). Ultra-thin multi-band polarization-insensitive microwave metamaterial absorber based on multiple-order responses using a single resonator structure. Materials.

[18] Okano Y., Ogino S., Ishikawa K. (2012). Development of optically transparent ultrathin microwave absorber for ultrahigh-frequency RF identification system. IEEE Trans. Microw. Theory Tech..

[19] Lee I.G., Yoon S.H., Lee J.S., Hong I.P. (2016). Design of wideband radar absorbing material with improved optical transmittance by using printed metal-mesh. Electron. Lett..

[20] Smith D.R., Vier D.C., Koschny T., Soukoulis C.M. (2005). Electromagnetic parameter retrieval from inhomogeneous metamaterials. Phys. Rev. E.

[21] Cui T.J., Smith D.R., Liu R.P. (2011). Metamaterials: Theory, Design and Applications.

[22] Baena J.D., Bonache J., Martin F., Sillero R.M., Falcone F., Lopetegi T., Laso M.A.G., Garcia-Garcia J., Gil I., Portillo M.F. (2005). Equivalent-circuit models for split-ring resonators and complementary split-ring resonators coupled to planar transmission lines. IEEE Trans. Microw. Theory Tech..

[23] Costa F., Monorchio A., Manara G. (2010). Analysis and design of ultra thin electromagnetic absorbers comprising resistively loaded high impedance surfaces. IEEE Trans. Antennas Propag..

[24] Pozar D.M. (2012). Microwave Engineering.

[25] Munk B.A. (2000). Frequency Selective Surfaces: Theory and Design.

[26] Langley R.J., Parker E.A. (1982). Equivalent-circuit model for arrays of square loops. Electron. Lett..

[27] Yu F., Wang J., Wang J.F., Ma H., Du H.L., Xu Z., Qu S.B. (2015). Reflective frequency selective surface based on low-permittivity dielectric metamaterials. Appl. Phys. Lett..

[28] Beadie G., Brindza M., Flynn R.A., Rosenberg A., Shirk J.S. (2015). Refractive index measurements of poly(methyl methacrylate) (PMMA) from 0.4–1.6 μm. Appl. Opt..

[29] Oh C.S., Lee S.M., Kim E.H., Lee E.W., Park L.S. (2012). Electro-optical properties of index matched ITO-PET film for touch panel application. Mol. Cryst. Liq. Cryst..

[30] Hecht E. (2002). Optics.

